# Supplementary material for: A comparison of model-free phase I dose escalation designs for dual-agent combination therapies
Source: Stat Methods Med Res. 2024 Jan 24;33(2):203–26. doi: 10.1177/09622802231220497 (PMC10928960; doi:10.1177/09622802231220497)
Supplement: sj-pdf-1-smm-10.1177_09622802231220497 - Supplemental material for A comparison of model-free phase I dose escalation designs for dual-agent combination therapies [file sj-pdf-1-smm-10.1177_09622802231220497.pdf]

A Comparison of Phase I Dose Escalation Designs for Combination Therapies: Online Supplementary Materials

1 Additional Graphs for Calibration Procedure

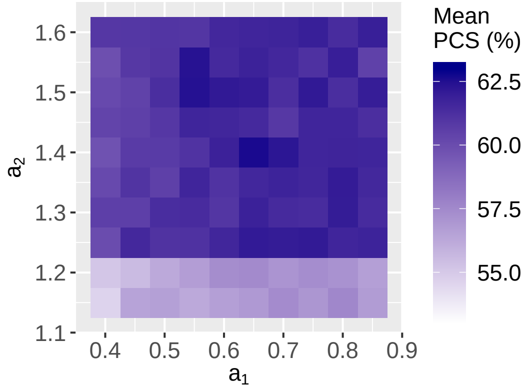

Figure 1: Calibration results for the BOIN design: Calibration of parameters a_1 and a_2 which define the interval affecting dose escalation in the BOIN design. We choose $a_1 = 0.65$ and $a_2 = 1.40$.

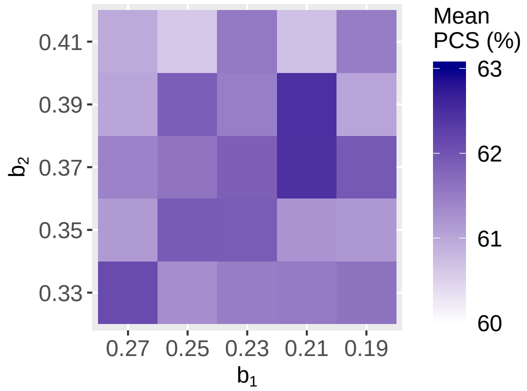

Figure 2: Calibration results for KEY: Calibration of parameters b_1 and b_2 which define the interval affecting dose escalation in KEY. We choose $b_1 = 0.21$ and $b_2 = 0.39$.

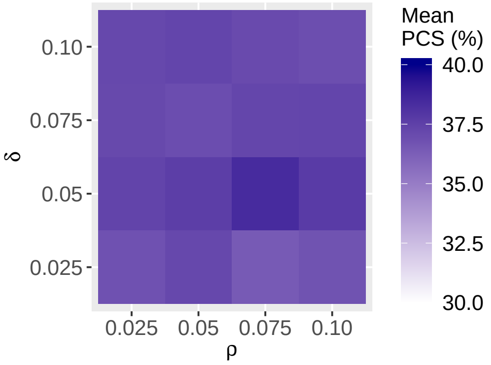

(a) Calibration results for `ss`=1/72.

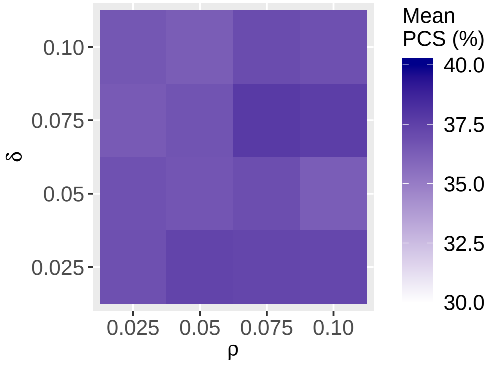

(b) Calibration results for `ss`=1/36.

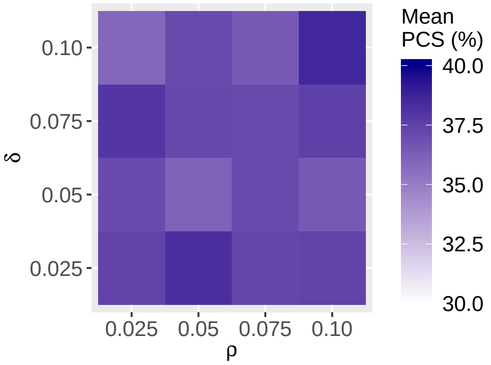

(c) Calibration results for `ss`=1/18.

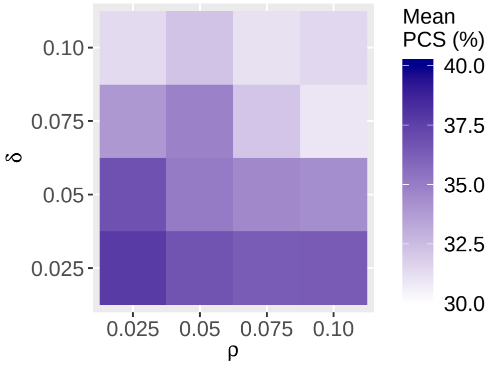

(d) Calibration results for `ss`=1/9.

Figure 3: Calibration results for the PIPE design: Values of $s = 1/18$, $\rho = 0.05$ and $\delta = 0.025$ define the operational priors.

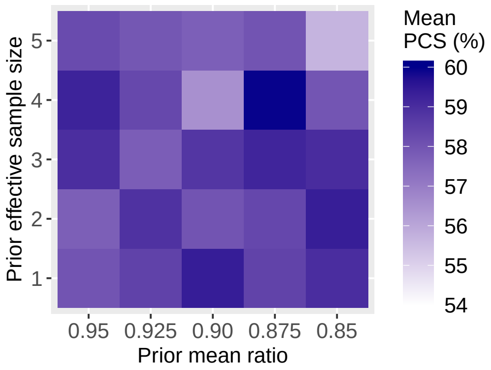

Figure 4: Calibration results for the SFD: Calibration of the prior mean ratios and effective sample sizes in the SFD. We choose values of $m = 0.875$ and $s = 4$.

2 Calibrating Model-Based Designs

2.1 BLRM

From our experience, the BLRM is very sensitive to its priors, which encourages the use of operational priors especially in cases where clinicians are uncertain about the prior toxicity of either agent. Its five parameters are assigned priors, but to make our calibration computationally feasible, we make a number of assumptions to reduce the number of hyper-parameters we specify to just five, so that the priors are in the form of Equations 1, 2 and 3. We assume the covariance between $\log(\alpha_1)$ and $\log(\beta_1)$ is zero, and that priors for $(\log(\alpha_1), \log(\beta_1))'$ and $(\log(\alpha_2), \log(\beta_2))'$ are equivalent. We further assume the mean of η is 0 since there is no knowledge before the trial begins on the interaction between the two drugs, which is the same approach taken in the original implementation [4]. This leaves us with two mean parameters c_1, c_2 and three variance parameters v_1, v_2, v_3 to specify in the operational priors.

$$\begin{pmatrix} \log(\alpha_1) \\ \log(\beta_1) \end{pmatrix} \sim \text{MVN}_2 \left(\begin{pmatrix} c_1 \\ c_2 \end{pmatrix}, \begin{pmatrix} v_1 & 0 \\ 0 & v_2 \end{pmatrix} \right) \tag{1}$$

$$\begin{pmatrix} \log(\alpha_2) \\ \log(\beta_2) \end{pmatrix} \sim \text{MVN}_2 \left(\begin{pmatrix} c_1 \\ c_2 \end{pmatrix}, \begin{pmatrix} v_1 & 0 \\ 0 & v_2 \end{pmatrix} \right) \tag{2}$$

$$\eta \sim N(0, v_3) \tag{3}$$

To calibrate these five parameters, we first fix $v_3 = 0.01$, which essentially prevents the design from modelling interactions. We run 500 simulations in each of the four scenarios, due to the computational demands, for different combinations of c_1, c_2, v_1, v_2 and v_3 from the sets $c_1 = \{-2.25, -2.5, -2.75\}$, $c_2 = \{0.6, 0.8, 1\}$, $v_1 = \{0.75, 1, 1.25\}$, $v_2 = \{0.20, 0.50, 1.0\}$ and $v_3 = \{0.03, 0.1, 0.5\}$. We choose these sets after some initial exploration of the design which suggested combinations of these values could result in high mean PCS.

The calibration results in the first stage identified that setting $c_1 = -2.5, c_2 = 0, v_1 = 1$, $v_2 = 0.5$ and $v_3 = 0.03$ in the priors in Equations 1, 2 and 3 yielded the highest mean PCS. In stage 2, the value of ϵ_{BLRM} is chosen to be 0.25, illustrated in Figure 5.

2.2 Partial Ordering Continual Reassessment Method

The POCRM design requires the specification of the following hyper-parameter parameter: μ, σ, $\tilde{q}_k$, and η. We fix $\mu = 0$, and calibrate σ, $\tilde{q}_k$ under the same scenarios and procedure as the competing design. For the skeleton values $\tilde{q}_k$, we find these by applying the function $g(\cdot)$ proposed by [1]. Specifically, the skeleton value at level k is given by $\tilde{q}_k = g(k, \phi, MTD_{loc}, \delta)$, where MTD_{loc} is the prior location of the MTD, and δ is the half-width of the indifference interval. We calibrate the POCRM design using $MTD_{loc} = 1, 2, \ldots, 9$, $\delta = 0.01, 0.015, 0.02, 0.025, 0.03, 0.035, 0.040$, and $\sigma^2 = 0.67, 1.34, 2.84, 4.26, 5.68$ where the value

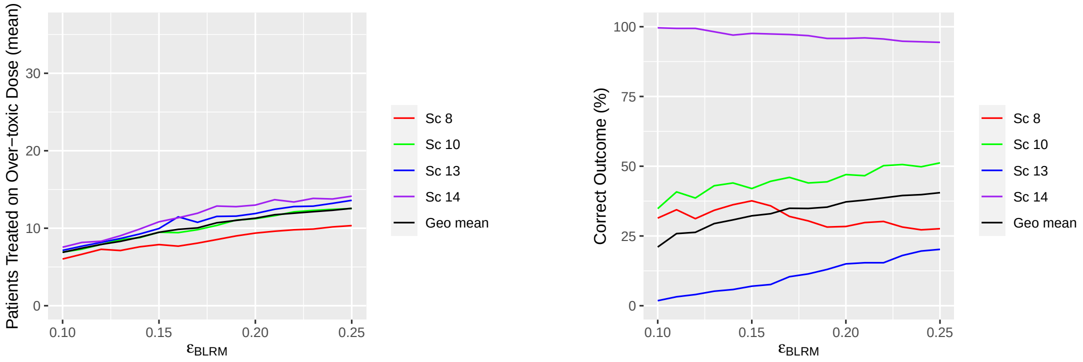

(a) Patients treated at overly toxic doses

(b) Proportion of correct selections

Figure 5: BLRM Prior Calibration: ϵ_{BLRM} value for EWOC.

of 1.34 is a commonly used value of variance, and 2.84 is the least informative prior variance [2]. Based on 1000 simulations for each combination of hyper-parameters, $\delta = 0.015$, $\sigma^2 = 2.84$, $MTD_{loc} = 3$ (dose d_{31}) were found to yield the highest geometric metric of the proportion of correct selections across four selected scenarios.

At the second stage of calibration, the various values of η to compute the lower bound of the confidence interval were tried. Specifically, $\eta = 0.50, 0.51, \ldots, 1.00$. The value of $\eta = 0.78$ was found to result in just over 85% of early terminations under Scenarios 14 and is used for the simulation study.

2.3 Logistic Model from Riviere

For the purposes of calibration, we fix the values for $p = (0.1, 0.2, 0.3)$, $q = (0.1, 0.2, 0.3)$ which are adequately spaced. The stage one hyper-parameters of the models parameters, β_0, β_1, β_2 are calibrated using the following prior distributions: $\beta_0 \sim \mathcal{N}(0, a)$, $\beta_1 \sim Gamma(b, b)$, $\beta_2 \sim Gamma(c, c)$, and with the value on the grids $a = 200, 400, 800, 1200, 1600, 2400, 3600, 4800, 7200$, $b = 0.25, 0.50, 1, 2.5, 5, 10, 20$, $c = 0.25, 0.50, 1, 2.5, 5, 10, 20$ that are constructed around the calibrated values used by [3]. The hyper-parameters $a = 4800$, $b = 0.25$, and $c = 2.5$ were found to yield the highest geometric mean of the proportion of correct selections across four selected scenarios.

At the second stage, $\epsilon_{logistic} = 0.80, 0.81, \ldots, 1$ were tried and $\epsilon_{logistic} = 0.84$ was taken forward as the value leading to at least 85% early terminations under Scenario 14.

3 Alternative BLRM prior

The BLRM showed aggressive performance in dose escalation and in selection in the simulation study, due to the values of the hyperparameters selected by the calibration procedure to maximise the PCS. Here we illustrate the difference in operating characteristics when using this

calibrated prior to when using an 'alternative' prior, whose hyperparameters have a more intuitive interpretation. This alternative prior is specified as in equations 1-3, with hyperparameter values $c_1 = log(0.25)$, $c_2 = 0$, $v_1 = 4$, $v_2 = 1$ & $v_3 = 0.03$, with $\epsilon_{\text{BLRM}} = 0.25$. These values are chosen so that doubling in dose was assumed to double odds of a DLT, to assume a median DLT rate of 20% at the reference dose, and to give a prior sample size of approximately 2 across all combinations.

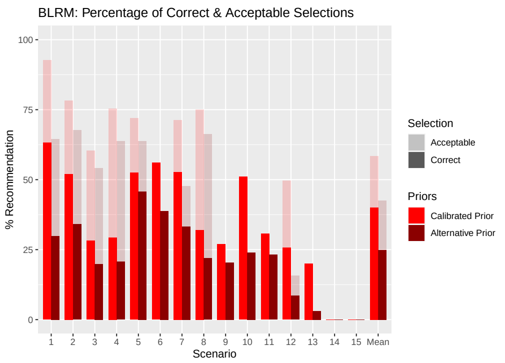

(a) Proportion of correct selections

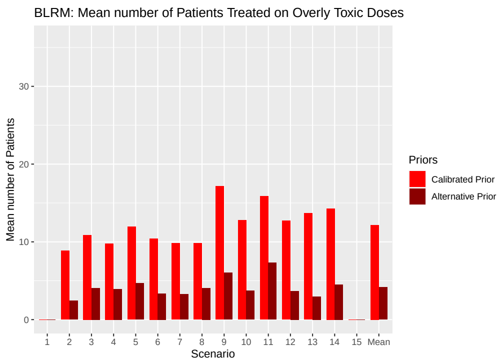

(b) Patients treated at overly toxic doses

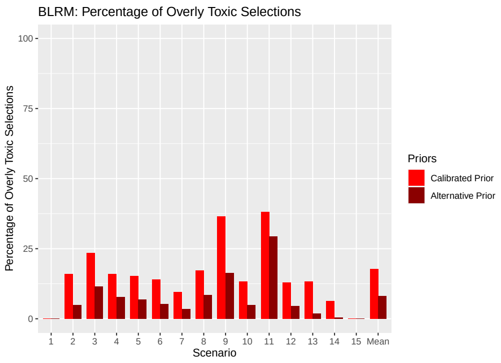

(c) Proportion of overly toxic selections

Figure 6: Operating characteristics of BLRM with both calibrated and alternative prior hyperparameters

It is clear from the results displayed in Figure 6 that the alternative prior is much less aggressive than the calibrated prior, with substantially fewer patients treated at unsafe dose, and a much lower proportion of simulations recommending overly toxic doses. However, this is of course accompanied by lower PCS and PAS in all scenarios, lower not only than the BLRM using the calibrated prior, but also lower than all other designs.

4 Additional Results Tables

Scenario 1

	d_1^B	d_2^B	d_3^B
d_1^A	0.2	0.5	6.7
d_2^A	0.4	3.0	<u>19.7</u>
d_3^A	7.2	<u>19.5</u>	<u>**41.6**</u>

Scenario 2

	d_1^B	d_2^B	d_3^B
d_1^A	0.1	0.8	15.7
d_2^A	1.2	<u>9.4</u>	<u>**25.7**</u>
d_3^A	<u>13.6</u>	<u>**22.2**</u>	10.1

Scenario 3

	d_1^B	d_2^B	d_3^B
d_1^A	0.0	0.2	8.9
d_2^A	0.7	4.5	<u>39.6</u>
d_3^A	<u>11.6</u>	<u>**22.1**</u>	12.3

Scenario 4

	d_1^B	d_2^B	d_3^B
d_1^A	0.1	1.1	14.8
d_2^A	1.6	<u>15.4</u>	<u>**34.2**</u>
d_3^A	<u>21.4</u>	9.1	1.4

Scenario 5

	d_1^B	d_2^B	d_3^B
d_1^A	0.4	2.5	39.0
d_2^A	<u>14.7</u>	<u>**25.5**</u>	10.5
d_3^A	5.6	1.6	0.1

Scenario 6

	d_1^B	d_2^B	d_3^B
d_1^A	0.6	9.7	<u>**19.1**</u>
d_2^A	9.0	<u>**22.8**</u>	8.1
d_3^A	<u>**18.6**</u>	7.2	0.7

Scenario 7

	d_1^B	d_2^B	d_3^B
d_1^A	1.6	<u>16.8</u>	6.0
d_2^A	12.8	<u>**27.9**</u>	3.6
d_3^A	<u>**21.4**</u>	5.3	0.3

Scenario 8

	d_1^B	d_2^B	d_3^B
d_1^A	0.1	1.6	<u>14.3</u>
d_2^A	5.0	<u>19.1</u>	<u>**33.5**</u>
d_3^A	<u>**14.8**</u>	8.2	1.9

Scenario 9

	d_1^B	d_2^B	d_3^B
d_1^A	5.0	20.4	<u>**30.6**</u>
d_2^A	<u>**16.1**</u>	11.9	3.3
d_3^A	7.0	1.9	0.2

Scenario 10

	d_1^B	d_2^B	d_3^B
d_1^A	17.9	<u>**25.9**</u>	6.0
d_2^A	<u>**25.2**</u>	8.6	0.7
d_3^A	6.2	0.7	0.0

Scenario 11

	d_1^B	d_2^B	d_3^B
d_1^A	1.1	3.1	60.8
d_2^A	<u>**21.1**</u>	8.2	0.9
d_3^A	4.5	0.2	0.0

Scenario 12

	d_1^B	d_2^B	d_3^B
d_1^A	<u>31.1</u>	<u>**31.6**</u>	7.0
d_2^A	8.0	3.8	0.5
d_3^A	0.2	0.0	0.0

Scenario 13

	d_1^B	d_2^B	d_3^B
d_1^A	<u>**37.7**</u>	7.2	1.2
d_2^A	7.2	1.4	0.2
d_3^A	1.1	0.1	0.0

Scenario 14

	d_1^B	d_2^B	d_3^B
d_1^A	10.7	2.0	0.1
d_2^A	1.6	0.1	0.1
d_3^A	0.1	0.0	0.0

Scenario 15

	d_1^B	d_2^B	d_3^B
d_1^A	0.1	0.1	3.0
d_2^A	0.2	0.5	3.2
d_3^A	3.4	3.4	82.1

Table 1: BOIN: Percentage of simulations selecting each dose combination True MTCs are in bold and 'acceptable' combinations are underlined.

Scenario 1

	d_1^B	d_2^B	d_3^B
d_1^A	3.6	2.3	2.4
d_2^A	2.2	3.0	5.0
d_3^A	2.5	5.1	**9.5**

Scenario 2

	d_1^B	d_2^B	d_3^B
d_1^A	3.6	2.4	4.1
d_2^A	2.5	4.1	**5.9**
d_3^A	3.7	**5.3**	3.9

Scenario 3

	d_1^B	d_2^B	d_3^B
d_1^A	3.2	1.9	2.9
d_2^A	2.4	3.2	8.2
d_3^A	3.5	**5.4**	5.3

Scenario 4

	d_1^B	d_2^B	d_3^B
d_1^A	3.6	2.4	3.9
d_2^A	2.6	5.0	**7.7**
d_3^A	5.0	3.8	1.8

Scenario 5

	d_1^B	d_2^B	d_3^B
d_1^A	3.6	2.9	8.8
d_2^A	5.3	**6.4**	4.4
d_3^A	2.7	1.5	0.5

Scenario 6

	d_1^B	d_2^B	d_3^B
d_1^A	4.4	4.4	**4.7**
d_2^A	4.2	**6.0**	2.9
d_3^A	**4.6**	2.9	0.6

Scenario 7

	d_1^B	d_2^B	d_3^B
d_1^A	4.7	5.4	2.8
d_2^A	4.9	**6.7**	1.9
d_3^A	**5.3**	2.5	0.4

Scenario 8

	d_1^B	d_2^B	d_3^B
d_1^A	3.6	2.6	3.7
d_2^A	3.2	5.5	**7.5**
d_3^A	**4.2**	3.5	1.8

Scenario 9

	d_1^B	d_2^B	d_3^B
d_1^A	5.7	6.4	**7.3**
d_2^A	**5.8**	4.2	1.8
d_3^A	2.3	1.1	0.3

Scenario 10

	d_1^B	d_2^B	d_3^B
d_1^A	8.9	**7.3**	2.6
d_2^A	**7.3**	3.3	0.6
d_3^A	2.6	0.5	0.1

Scenario 11

	d_1^B	d_2^B	d_3^B
d_1^A	4.2	3.4	13.6
d_2^A	**6.3**	3.9	1.6
d_3^A	2.3	0.6	0.1

Scenario 12

	d_1^B	d_2^B	d_3^B
d_1^A	11.9	**8.4**	3.0
d_2^A	4.5	1.9	0.4
d_3^A	0.5	0.2	0.0

Scenario 13

	d_1^B	d_2^B	d_3^B
d_1^A	**13.7**	3.7	0.7
d_2^A	3.7	0.8	0.1
d_3^A	0.8	0.1	0.0

Scenario 14

	d_1^B	d_2^B	d_3^B
d_1^A	9.0	1.5	0.2
d_2^A	1.3	0.2	0.0
d_3^A	0.2	0.0	0.0

Scenario 15

	d_1^B	d_2^B	d_3^B
d_1^A	4.1	2.1	1.6
d_2^A	2.1	2.0	2.3
d_3^A	1.6	2.3	16.5

Table 2: BOIN: Mean number of patients assigned to each dose combination. True MTCs are in bold and ‘acceptable’ combinations are underlined.

Scenario 1

	d_1^B	d_2^B	d_3^B
d_1^A	0.1	0.8	5.5
d_2^A	0.5	4.1	19.2
d_3^A	6.8	19.6	**43.1**

Scenario 2

	d_1^B	d_2^B	d_3^B
d_1^A	0.1	0.5	12.7
d_2^A	2.0	14.0	**24.8**
d_3^A	13.3	**22.7**	9.8

Scenario 3

	d_1^B	d_2^B	d_3^B
d_1^A	0.1	0.2	5.0
d_2^A	1.4	6.8	37.7
d_3^A	11.2	**24.8**	12.9

Scenario 4

	d_1^B	d_2^B	d_3^B
d_1^A	0.1	1.0	13.5
d_2^A	1.8	18.7	**33.1**
d_3^A	19.3	11.2	1.2

Scenario 5

	d_1^B	d_2^B	d_3^B
d_1^A	0.1	2.2	31.9
d_2^A	15.7	**31.1**	9.8
d_3^A	6.6	2.4	0.1

Scenario 6

	d_1^B	d_2^B	d_3^B
d_1^A	1.1	10.7	**16.2**
d_2^A	10.5	**24.8**	7.7
d_3^A	**19.7**	7.1	0.5

Scenario 7

	d_1^B	d_2^B	d_3^B
d_1^A	1.4	18.4	6.5
d_2^A	11.8	**30.5**	2.9
d_3^A	**21.8**	5.2	0.1

Scenario 8

	d_1^B	d_2^B	d_3^B
d_1^A	0.0	2.1	13.4
d_2^A	3.8	21.4	**31.1**
d_3^A	**17.5**	8.5	1.8

Scenario 9

	d_1^B	d_2^B	d_3^B
d_1^A	3.5	19.3	**29.7**
d_2^A	**20.6**	12.5	3.0
d_3^A	7.3	2.1	0.1

Scenario 10

	d_1^B	d_2^B	d_3^B
d_1^A	16.4	**29.0**	6.2
d_2^A	**28.3**	7.1	0.7
d_3^A	6.2	0.6	0.0

Scenario 11

	d_1^B	d_2^B	d_3^B
d_1^A	1.2	3.4	52.5
d_2^A	**25.9**	10.5	0.8
d_3^A	5.3	0.2	0.0

Scenario 12

	d_1^B	d_2^B	d_3^B
d_1^A	33.5	**33.3**	6.1
d_2^A	11.4	3.0	0.2
d_3^A	0.3	0.0	0.0

Scenario 13

	d_1^B	d_2^B	d_3^B
d_1^A	**43.1**	7.0	1.1
d_2^A	6.6	1.1	0.2
d_3^A	1.0	0.1	0.0

Scenario 14

	d_1^B	d_2^B	d_3^B
d_1^A	11.3	1.2	0.1
d_2^A	1.3	0.1	0.1
d_3^A	0.1	0.0	0.0

Scenario 15

	d_1^B	d_2^B	d_3^B
d_1^A	0.8	0.2	1.6
d_2^A	0.4	1.1	2.8
d_3^A	2.2	2.7	87.0

Table 3: Keyboard: Percentage of simulations selecting each dose combination True MTCs are in bold and 'acceptable' combinations are underlined.

Scenario 1

	d_1^B	d_2^B	d_3^B
d_1^A	3.7	2.6	2.3
d_2^A	2.5	3.3	<u>4.7</u>
d_3^A	2.4	<u>4.9</u>	<u>**9.5**</u>

Scenario 2

	d_1^B	d_2^B	d_3^B
d_1^A	3.8	2.6	3.2
d_2^A	3.0	<u>5.1</u>	<u>**5.7**</u>
d_3^A	<u>3.7</u>	<u>**5.1**</u>	3.8

Scenario 3

	d_1^B	d_2^B	d_3^B
d_1^A	3.3	2.1	2.1
d_2^A	2.7	4.0	<u>7.7</u>
d_3^A	<u>3.1</u>	<u>**5.8**</u>	5.2

Scenario 4

	d_1^B	d_2^B	d_3^B
d_1^A	3.7	2.6	3.5
d_2^A	2.8	<u>5.7</u>	<u>**7.0**</u>
d_3^A	<u>4.7</u>	4.1	1.8

Scenario 5

	d_1^B	d_2^B	d_3^B
d_1^A	3.6	2.8	6.6
d_2^A	<u>6.1</u>	<u>**7.7**</u>	4.2
d_3^A	2.8	1.6	0.5

Scenario 6

	d_1^B	d_2^B	d_3^B
d_1^A	5.0	4.8	<u>**3.9**</u>
d_2^A	4.8	<u>**6.7**</u>	2.6
d_3^A	<u>**4.5**</u>	2.6	0.5

Scenario 7

	d_1^B	d_2^B	d_3^B
d_1^A	5.3	<u>6.1</u>	2.8
d_2^A	5.1	<u>**6.9**</u>	1.7
d_3^A	<u>**4.9**</u>	2.4	0.3

Scenario 8

	d_1^B	d_2^B	d_3^B
d_1^A	3.8	2.8	<u>3.3</u>
d_2^A	3.5	<u>6.2</u>	<u>**6.5**</u>
d_3^A	<u>**4.4**</u>	3.6	1.7

Scenario 9

	d_1^B	d_2^B	d_3^B
d_1^A	6.4	6.2	<u>**6.2**</u>
d_2^A	<u>**7.2**</u>	4.3	1.5
d_3^A	2.2	1.0	0.3

Scenario 10

	d_1^B	d_2^B	d_3^B
d_1^A	9.9	<u>**8.3**</u>	2.2
d_2^A	<u>**7.8**</u>	3.0	0.5
d_3^A	2.2	0.4	0.1

Scenario 11

	d_1^B	d_2^B	d_3^B
d_1^A	4.5	3.7	9.8
d_2^A	<u>**8.5**</u>	4.9	1.8
d_3^A	2.1	0.6	0.1

Scenario 12

	d_1^B	d_2^B	d_3^B
d_1^A	<u>14.0</u>	<u>**8.3**</u>	2.4
d_2^A	5.2	1.7	0.3
d_3^A	0.5	0.1	0.0

Scenario 13

	d_1^B	d_2^B	d_3^B
d_1^A	<u>**16.4**</u>	3.6	0.6
d_2^A	3.7	0.7	0.1
d_3^A	0.5	0.1	0.0

Scenario 14

	d_1^B	d_2^B	d_3^B
d_1^A	10.9	1.2	0.1
d_2^A	1.1	0.2	0.0
d_3^A	0.1	0.0	0.0

Scenario 15

	d_1^B	d_2^B	d_3^B
d_1^A	4.8	2.4	1.4
d_2^A	2.3	2.3	2.3
d_3^A	1.4	2.2	16.5

Table 4: Keyboard: Mean number of patients assigned to each dose combination. True MTCs are in bold and 'acceptable' combinations are underlined.

Scenario 1

	d_1^B	d_2^B	d_3^B
d_1^A	0.0	2.0	9.6
d_2^A	1.6	4.0	15.2
d_3^A	11.1	14.3	**41.5**

Scenario 2

	d_1^B	d_2^B	d_3^B
d_1^A	0.0	2.5	13.4
d_2^A	2.6	12.4	**20.8**
d_3^A	17.0	**17.2**	13.4

Scenario 3

	d_1^B	d_2^B	d_3^B
d_1^A	0.0	0.9	8.1
d_2^A	1.7	4.7	24.3
d_3^A	17.9	**20.3**	22.0

Scenario 4

	d_1^B	d_2^B	d_3^B
d_1^A	0.0	2.5	14.6
d_2^A	2.7	15.4	**23.2**
d_3^A	22.9	14.6	3.5

Scenario 5

	d_1^B	d_2^B	d_3^B
d_1^A	0.0	2.0	18.6
d_2^A	20.4	**30.3**	16.4
d_3^A	10.1	1.8	0.3

Scenario 6

	d_1^B	d_2^B	d_3^B
d_1^A	0.2	9.3	**18.2**
d_2^A	9.5	**26.2**	7.5
d_3^A	**18.4**	6.8	0.5

Scenario 7

	d_1^B	d_2^B	d_3^B
d_1^A	0.5	15.6	9.8
d_2^A	10.2	**29.4**	2.6
d_3^A	**22.5**	6.2	0.1

Scenario 8

	d_1^B	d_2^B	d_3^B
d_1^A	0.0	2.0	14.1
d_2^A	3.3	18.9	2**4.5**
d_3^A	**22.9**	10.8	3.0

Scenario 9

	d_1^B	d_2^B	d_3^B
d_1^A	0.8	13.6	**25.6**
d_2^A	**27.6**	19.3	2.6
d_3^A	5.8	1.2	0.1

Scenario 10

	d_1^B	d_2^B	d_3^B
d_1^A	6.7	**31.3**	6.2
d_2^A	**30.5**	9.6	0.3
d_3^A	6.5	0.1	0.0

Scenario 11

	d_1^B	d_2^B	d_3^B
d_1^A	0.0	2.5	23.6
d_2^A	**51.2**	15.6	2.5
d_3^A	4.0	0.5	0.0

Scenario 12

	d_1^B	d_2^B	d_3^B
d_1^A	17.4	**34.4**	7.5
d_2^A	18.6	2.8	0.1
d_3^A	0.2	0.1	0.0

Scenario 13

	d_1^B	d_2^B	d_3^B
d_1^A	**24.4**	9.2	0.8
d_2^A	11.3	0.7	0.0
d_3^A	0.9	0.0	0.0

Scenario 14

	d_1^B	d_2^B	d_3^B
d_1^A	5.7	1.8	0.2
d_2^A	1.8	0.1	0.0
d_3^A	0.2	0.1	0.0

Scenario 15

	d_1^B	d_2^B	d_3^B
d_1^A	0.1	2.4	5.1
d_2^A	1.9	1.1	3.1
d_3^A	5.5	3.0	74.5

Table 5: Surface Free: Percentage of simulations selecting each dose combination True MTCs are in bold and 'acceptable' combinations are underlined.

Scenario 1

	d_1^B	d_2^B	d_3^B
d_1^A	3.4	3.0	3.6
d_2^A	2.6	5.1	<u>4.7</u>
d_3^A	3.9	<u>4.6</u>	<u>**4.9**</u>

Scenario 2

	d_1^B	d_2^B	d_3^B
d_1^A	3.4	3.2	4.1
d_2^A	3.0	<u>6.7</u>	<u>**4.8**</u>
d_3^A	<u>4.5</u>	<u>**4.2**</u>	1.9

Scenario 3

	d_1^B	d_2^B	d_3^B
d_1^A	3.2	2.4	3.5
d_2^A	2.4	5.6	<u>6.1</u>
d_3^A	<u>4.6</u>	<u>**5.0**</u>	3.3

Scenario 4

	d_1^B	d_2^B	d_3^B
d_1^A	3.4	3.2	4.2
d_2^A	3.0	<u>7.1</u>	<u>**5.1**</u>
d_3^A	<u>5.1</u>	3.9	0.8

Scenario 5

	d_1^B	d_2^B	d_3^B
d_1^A	3.2	3.6	7.0
d_2^A	<u>5.0</u>	<u>**9.5**</u>	3.5
d_3^A	3.2	0.8	0.1

Scenario 6

	d_1^B	d_2^B	d_3^B
d_1^A	4.1	5.5	<u>**4.3**</u>
d_2^A	5.1	<u>**8.3**</u>	1.7
d_3^A	<u>**4.3**</u>	1.6	0.1

Scenario 7

	d_1^B	d_2^B	d_3^B
d_1^A	4.4	<u>6.3</u>	3.0
d_2^A	5.6	<u>**8.3**</u>	0.6
d_3^A	<u>**5.2**</u>	1.5	0.0

Scenario 8

	d_1^B	d_2^B	d_3^B
d_1^A	3.4	3.3	<u>4.6</u>
d_2^A	3.2	<u>7.9</u>	<u>**4.9**</u>
d_3^A	<u>**5.0**</u>	2.9	0.5

Scenario 9

	d_1^B	d_2^B	d_3^B
d_1^A	4.9	7.7	<u>**5.8**</u>
d_2^A	<u>**7.0**</u>	6.1	0.7
d_3^A	2.1	0.4	0.0

Scenario 10

	d_1^B	d_2^B	d_3^B
d_1^A	8.3	<u>**8.6**</u>	2.0
d_2^A	<u>**8.7**</u>	3.5	0.1
d_3^A	2.1	0.1	0.0

Scenario 11

	d_1^B	d_2^B	d_3^B
d_1^A	3.2	4.7	10.6
d_2^A	<u>**7.0**</u>	7.2	1.0
d_3^A	2.0	0.3	0.0

Scenario 12

	d_1^B	d_2^B	d_3^B
d_1^A	<u>11.1</u>	<u>**9.7**</u>	2.2
d_2^A	5.7	1.7	0.0
d_3^A	0.4	0.0	0.0

Scenario 13

	d_1^B	d_2^B	d_3^B
d_1^A	<u>**12.7**</u>	4.4	0.5
d_2^A	4.6	0.6	0.0
d_3^A	0.5	0.0	0.0

Scenario 14

	d_1^B	d_2^B	d_3^B
d_1^A	8.5	1.9	0.1
d_2^A	1.9	0.2	0.0
d_3^A	0.2	0.0	0.0

Scenario 15

	d_1^B	d_2^B	d_3^B
d_1^A	4.0	3.0	2.8
d_2^A	2.6	3.9	3.4
d_3^A	2.9	3.4	9.0

Table 6: Surface Free : Mean number of patients assigned to each dose combination. True MTCs are in bold and 'acceptable' combinations are underlined.

Scenario 1

	d_1^B	d_2^B	d_3^B
d_1^A	0.2	1.6	16.6
d_2^A	1.4	8.2	23.6
d_3^A	16.6	24.9	**5.5**

Scenario 2

	d_1^B	d_2^B	d_3^B
d_1^A	0.0	3.5	25.4
d_2^A	5.0	16.9	**13.1**
d_3^A	22.2	**11.9**	0.6

Scenario 3

	d_1^B	d_2^B	d_3^B
d_1^A	0.1	0.8	16.8
d_2^A	2.4	11.4	26.2
d_3^A	22.8	**18.1**	0.8

Scenario 4

	d_1^B	d_2^B	d_3^B
d_1^A	0.1	3.4	25.2
d_2^A	5.6	20.4	**11.6**
d_3^A	27.2	5.3	0.1

Scenario 5

	d_1^B	d_2^B	d_3^B
d_1^A	0.2	6.3	36.9
d_2^A	20.7	**21.1**	2.5
d_3^A	10.8	0.5	0.0

Scenario 6

	d_1^B	d_2^B	d_3^B
d_1^A	1.5	16.4	**20.5**
d_2^A	17.9	**19.1**	1.6
d_3^A	**20.1**	1.8	0.0

Scenario 7

	d_1^B	d_2^B	d_3^B
d_1^A	3.0	23.4	9.0
d_2^A	21.6	**19.8**	0.3
d_3^A	**21.2**	0.8	0.0

Scenario 8

	d_1^B	d_2^B	d_3^B
d_1^A	0.3	4.7	25.0
d_2^A	7.7	25.4	**10.6**
d_3^A	**21.6**	3.0	0.0

Scenario 9

	d_1^B	d_2^B	d_3^B
d_1^A	6.5	33.7	**23.1**
d_2^A	**19.1**	7.4	0.3
d_3^A	8.8	0.3	0.0

Scenario 10

	d_1^B	d_2^B	d_3^B
d_1^A	30.7	**26.6**	4.6
d_2^A	**25.3**	2.6	0.0
d_3^A	4.8	0.2	0.0

Scenario 11

	d_1^B	d_2^B	d_3^B
d_1^A	0.8	5.5	51.7
d_2^A	**27.4**	6.2	0.1
d_3^A	7.9	0.1	0.0

Scenario 12

	d_1^B	d_2^B	d_3^B
d_1^A	44.1	**30.6**	2.8
d_2^A	7.5	1.1	0.0
d_3^A	0.3	0.0	0.0

Scenario 13

	d_1^B	d_2^B	d_3^B
d_1^A	**39.0**	6.0	0.9
d_2^A	5.7	0.8	0.0
d_3^A	0.8	0.0	0.0

Scenario 14

	d_1^B	d_2^B	d_3^B
d_1^A	8.2	1.8	0.3
d_2^A	1.9	0.1	0.0
d_3^A	0.2	0.0	0.0

Scenario 15

	d_1^B	d_2^B	d_3^B
d_1^A	0.0	0.3	8.0
d_2^A	0.3	1.4	19.8
d_3^A	7.7	21.9	38.9

Table 7: PIPE: Percentage of simulations selecting each dose combination True MTCs are in bold and 'acceptable' combinations are underlined.

Scenario 1

	d_1^B	d_2^B	d_3^B
d_1^A	3.1	1.9	5.3
d_2^A	2.0	6.2	5.4
d_3^A	5.3	5.4	**1.4**

Scenario 2

	d_1^B	d_2^B	d_3^B
d_1^A	3.0	2.5	6.9
d_2^A	2.5	7.5	**3.7**
d_3^A	6.2	**3.4**	0.3

Scenario 3

	d_1^B	d_2^B	d_3^B
d_1^A	3.0	1.9	5.8
d_2^A	1.9	6.8	5.9
d_3^A	5.5	**4.7**	0.6

Scenario 4

	d_1^B	d_2^B	d_3^B
d_1^A	3.0	2.4	6.9
d_2^A	2.5	8.0	**3.5**
d_3^A	6.8	2.7	0.1

Scenario 5

	d_1^B	d_2^B	d_3^B
d_1^A	3.1	4.1	10.5
d_2^A	4.2	**7.7**	1.6
d_3^A	4.3	0.5	0.0

Scenario 6

	d_1^B	d_2^B	d_3^B
d_1^A	3.4	4.9	**6.5**
d_2^A	4.9	**7.7**	1.1
d_3^A	**6.4**	1.0	0.0

Scenario 7

	d_1^B	d_2^B	d_3^B
d_1^A	3.6	5.8	4.6
d_2^A	6.0	**7.8**	0.4
d_3^A	**7.0**	0.8	0.0

Scenario 8

	d_1^B	d_2^B	d_3^B
d_1^A	3.1	2.8	7.3
d_2^A	3.1	8.7	**2.9**
d_3^A	**6.1**	1.9	0.1

Scenario 9

	d_1^B	d_2^B	d_3^B
d_1^A	4.4	8.4	**7.5**
d_2^A	**5.8**	5.3	0.4
d_3^A	3.9	0.3	0.0

Scenario 10

	d_1^B	d_2^B	d_3^B
d_1^A	7.8	**8.2**	3.5
d_2^A	**8.2**	3.9	0.1
d_3^A	3.6	0.1	0.0

Scenario 11

	d_1^B	d_2^B	d_3^B
d_1^A	3.1	5.0	13.8
d_2^A	**4.7**	5.3	0.6
d_3^A	3.3	0.2	0.0

Scenario 12

	d_1^B	d_2^B	d_3^B
d_1^A	11.0	**10.0**	3.2
d_2^A	6.1	2.6	0.0
d_3^A	1.5	0.0	0.0

Scenario 13

	d_1^B	d_2^B	d_3^B
d_1^A	**13.5**	5.7	1.7
d_2^A	5.5	2.0	0.0
d_3^A	1.6	0.0	0.0

Scenario 14

	d_1^B	d_2^B	d_3^B
d_1^A	10.8	3.4	0.8
d_2^A	3.4	1.0	0.0
d_3^A	0.8	0.0	0.0

Scenario 15

	d_1^B	d_2^B	d_3^B
d_1^A	3.1	1.7	3.9
d_2^A	1.7	4.6	5.7
d_3^A	3.8	5.8	5.7

Table 8: PIPE: Mean number of patients assigned to each dose combination. True MTCs are in bold and 'acceptable' combinations are underlined.

Scenario 1

	d_1^B	d_2^B	d_3^B
d_1^A	1.1	1.4	26.9
d_2^A	2.2	5.2	<u>27.7</u>
d_3^A	8.0	<u>16.8</u>	<u>**9.6**</u>

Scenario 2

	d_1^B	d_2^B	d_3^B
d_1^A	1.8	2.2	24.1
d_2^A	3.7	<u>13.2</u>	<u>**22.8**</u>
d_3^A	<u>13.2</u>	<u>**15.4**</u>	2.7

Scenario 3

	d_1^B	d_2^B	d_3^B
d_1^A	0.3	1.0	19.4
d_2^A	2.9	8.3	<u>30.5</u>
d_3^A	<u>15.2</u>	<u>**17.4**</u>	4.5

Scenario 4

	d_1^B	d_2^B	d_3^B
d_1^A	2.4	2.3	24.2
d_2^A	4.8	<u>14.4</u>	<u>**19.6**</u>
d_3^A	<u>20.4</u>	10.2	0.6

Scenario 5

	d_1^B	d_2^B	d_3^B
d_1^A	1.4	3.2	36.0
d_2^A	<u>24.2</u>	<u>**16.9**</u>	6.6
d_3^A	8.7	2.5	0.1

Scenario 6

	d_1^B	d_2^B	d_3^B
d_1^A	6.8	9.5	<u>**25.1**</u>
d_2^A	10.9	<u>**15.3**</u>	5.5
d_3^A	<u>**14.4**</u>	7.8	0.5

Scenario 7

	d_1^B	d_2^B	d_3^B
d_1^A	8.5	<u>15.0</u>	12.1
d_2^A	11.7	<u>**20.1**</u>	3.6
d_3^A	<u>**17.8**</u>	6.9	0.4

Scenario 8

	d_1^B	d_2^B	d_3^B
d_1^A	1.9	4.4	<u>29.9</u>
d_2^A	5.1	<u>16.1</u>	<u>**16.8**</u>
d_3^A	<u>**17.4**</u>	7.1	0.3

Scenario 9

	d_1^B	d_2^B	d_3^B
d_1^A	10.1	15.7	<u>**30.8**</u>
d_2^A	<u>**22.6**</u>	6.6	1.4
d_3^A	5.8	2.6	0.2

Scenario 10

	d_1^B	d_2^B	d_3^B
d_1^A	25.7	<u>**18.4**</u>	10.8
d_2^A	<u>**21.8**</u>	6.5	0.6
d_3^A	4.2	2.0	0.1

Scenario 11

	d_1^B	d_2^B	d_3^B
d_1^A	2.0	2.5	47.0
d_2^A	<u>**32.1**</u>	6.8	0.9
d_3^A	6.4	1.8	0.0

Scenario 12

	d_1^B	d_2^B	d_3^B
d_1^A	<u>38.3</u>	<u>**21.2**</u>	11.2
d_2^A	11.1	0.9	0.0
d_3^A	0.3	0.1	0.0

Scenario 13

	d_1^B	d_2^B	d_3^B
d_1^A	<u>**43.8**</u>	6.8	1.8
d_2^A	7.2	0.9	0.1
d_3^A	0.8	0.6	0.0

Scenario 14

	d_1^B	d_2^B	d_3^B
d_1^A	23.9	1.4	0.1
d_2^A	2.0	0.1	0.0
d_3^A	0.3	0.1	0.0

Scenario 15

	d_1^B	d_2^B	d_3^B
d_1^A	2.1	0.5	22.4
d_2^A	1.4	1.1	20.4
d_3^A	4.0	16.0	28.1

Table 9: Waterfall: Percentage of simulations selecting each dose combination True MTCs are in bold and 'acceptable' combinations are underlined.

Scenario 1

	d_1^B	d_2^B	d_3^B
d_1^A	3.8	0.6	7.8
d_2^A	4.5	1.6	6.4
d_3^A	4.4	3.4	**1.7**

Scenario 2

	d_1^B	d_2^B	d_3^B
d_1^A	3.8	0.7	7.3
d_2^A	4.9	2.7	**4.7**
d_3^A	5.0	**3.1**	0.9

Scenario 3

	d_1^B	d_2^B	d_3^B
d_1^A	3.5	0.4	8.2
d_2^A	5.0	2.2	5.8
d_3^A	5.1	**3.2**	1.0

Scenario 4

	d_1^B	d_2^B	d_3^B
d_1^A	3.8	0.7	7.2
d_2^A	4.9	3.2	**4.0**
d_3^A	5.6	3.0	0.4

Scenario 5

	d_1^B	d_2^B	d_3^B
d_1^A	4.6	2.0	6.2
d_2^A	8.0	**3.0**	1.5
d_3^A	4.1	0.8	0.1

Scenario 6

	d_1^B	d_2^B	d_3^B
d_1^A	4.5	1.8	**4.5**
d_2^A	5.7	**3.1**	2.1
d_3^A	**4.7**	1.8	0.2

Scenario 7

	d_1^B	d_2^B	d_3^B
d_1^A	4.5	2.5	3.1
d_2^A	5.7	**3.2**	1.9
d_3^A	**4.8**	1.7	0.1

Scenario 8

	d_1^B	d_2^B	d_3^B
d_1^A	3.8	1.0	6.9
d_2^A	5.9	3.5	**3.8**
d_3^A	**5.5**	2.2	0.3

Scenario 9

	d_1^B	d_2^B	d_3^B
d_1^A	6.6	3.1	**3.6**
d_2^A	**6.3**	1.5	0.6
d_3^A	2.8	0.7	0.1

Scenario 10

	d_1^B	d_2^B	d_3^B
d_1^A	6.4	**2.8**	1.7
d_2^A	**6.0**	1.4	0.4
d_3^A	2.4	0.4	0.0

Scenario 11

	d_1^B	d_2^B	d_3^B
d_1^A	6.3	2.6	5.2
d_2^A	**7.3**	1.4	0.4
d_3^A	3.0	0.5	0.0

Scenario 12

	d_1^B	d_2^B	d_3^B
d_1^A	8.0	**2.9**	1.4
d_2^A	4.7	0.4	0.1
d_3^A	0.8	0.0	0.0

Scenario 13

	d_1^B	d_2^B	d_3^B
d_1^A	**6.1**	1.2	0.3
d_2^A	3.1	0.3	0.1
d_3^A	0.6	0.1	0.0

Scenario 14

	d_1^B	d_2^B	d_3^B
d_1^A	2.9	0.3	0.1
d_2^A	1.2	0.1	0.0
d_3^A	0.2	0.0	0.0

Scenario 15

	d_1^B	d_2^B	d_3^B
d_1^A	4.1	0.4	8.0
d_2^A	4.0	0.6	7.4
d_3^A	3.5	3.0	2.5

Table 10: Waterfall: Mean number of patients assigned to each dose combination. True MTCs are in bold and 'acceptable' combinations are underlined.

Scenario 1

	d_1^B	d_2^B	d_3^B
d_1^A	0.0	0.3	0.3
d_2^A	0.2	5.4	<u>14.3</u>
d_3^A	0.0	<u>15.2</u>	**<u>63.2</u>**

Scenario 2

	d_1^B	d_2^B	d_3^B
d_1^A	0.2	1.7	1.5
d_2^A	1.4	<u>25.1</u>	**<u>26.6</u>**
d_3^A	<u>1.1</u>	**<u>25.4</u>**	15.9

Scenario 3

	d_1^B	d_2^B	d_3^B
d_1^A	0.0	0.2	0.4
d_2^A	0.5	13.4	<u>31.3</u>
d_3^A	<u>0.8</u>	**<u>28.2</u>**	23.5

Scenario 4

	d_1^B	d_2^B	d_3^B
d_1^A	0.1	2.1	1.1
d_2^A	3.5	<u>44.9</u>	**<u>29.3</u>**
d_3^A	<u>1.1</u>	13.5	2.4

Scenario 5

	d_1^B	d_2^B	d_3^B
d_1^A	0.3	5.0	4.7
d_2^A	<u>19.6</u>	**<u>52.5</u>**	12.8
d_3^A	0.6	1.7	0.1

Scenario 6

	d_1^B	d_2^B	d_3^B
d_1^A	1.0	13.3	**<u>2.8</u>**
d_2^A	12.2	**<u>52.1</u>**	7.8
d_3^A	**<u>1.1</u>**	5.8	0.3

Scenario 7

	d_1^B	d_2^B	d_3^B
d_1^A	2.0	<u>18.6</u>	1.2
d_2^A	12.8	**<u>51.8</u>**	3.2
d_3^A	**<u>0.9</u>**	4.8	0.2

Scenario 8

	d_1^B	d_2^B	d_3^B
d_1^A	0.1	2.3	<u>1.3</u>
d_2^A	3.6	<u>41.8</u>	**<u>30.8</u>**
d_3^A	**<u>1.2</u>**	13.3	3.8

Scenario 9

	d_1^B	d_2^B	d_3^B
d_1^A	5.8	23.9	**<u>3.5</u>**
d_2^A	**<u>23.6</u>**	29.8	4.7
d_3^A	0.5	1.5	0.1

Scenario 10

	d_1^B	d_2^B	d_3^B
d_1^A	19.4	**<u>28.4</u>**	0.6
d_2^A	**<u>22.6</u>**	11.7	0.6
d_3^A	0.2	0.1	0.1

Scenario 11

	d_1^B	d_2^B	d_3^B
d_1^A	1.6	12.8	10.7
d_2^A	**<u>30.6</u>**	34.2	3.8
d_3^A	0.1	0.1	0.1

Scenario 12

	d_1^B	d_2^B	d_3^B
d_1^A	<u>23.9</u>	**<u>25.7</u>**	1.0
d_2^A	7.0	4.7	0.3
d_3^A	0.0	0.0	0.0

Scenario 13

	d_1^B	d_2^B	d_3^B
d_1^A	**<u>20.1</u>**	5.7	0.1
d_2^A	5.4	1.9	0.1
d_3^A	0.1	0.1	0.0

Scenario 14

	d_1^B	d_2^B	d_3^B
d_1^A	4.1	1.1	0.0
d_2^A	0.8	0.3	0.0
d_3^A	0.0	0.0	0.0

Scenario 15

	d_1^B	d_2^B	d_3^B
d_1^A	0.0	0.1	0.0
d_2^A	0.0	0.6	0.8
d_3^A	0.0	0.3	97.5

Table 11: BLRM: Percentage of simulations selecting each dose combination True MTCs are in bold and ‘acceptable’ combinations are underlined.

Scenario 1

	d_1^B	d_2^B	d_3^B
d_1^A	3.0	1.6	1.2
d_2^A	1.7	2.1	4.1
d_3^A	1.2	4.1	**17.0**

Scenario 2

	d_1^B	d_2^B	d_3^B
d_1^A	3.1	1.9	1.4
d_2^A	2.0	5.4	**6.0**
d_3^A	1.3	**5.9**	8.9

Scenario 3

	d_1^B	d_2^B	d_3^B
d_1^A	3.0	1.6	1.3
d_2^A	1.7	3.5	6.5
d_3^A	1.4	**5.9**	10.9

Scenario 4

	d_1^B	d_2^B	d_3^B
d_1^A	3.2	2.1	1.4
d_2^A	2.4	8.7	**7.0**
d_3^A	1.3	4.9	4.9

Scenario 5

	d_1^B	d_2^B	d_3^B
d_1^A	3.6	3.1	2.2
d_2^A	4.4	**10.4**	5.6
d_3^A	1.3	2.5	2.6

Scenario 6

	d_1^B	d_2^B	d_3^B
d_1^A	3.8	4.1	**1.5**
d_2^A	3.7	**10.7**	4.1
d_3^A	**1.1**	3.7	2.6

Scenario 7

	d_1^B	d_2^B	d_3^B
d_1^A	4.1	4.8	1.2
d_2^A	4.2	**11.2**	3.0
d_3^A	**1.2**	3.6	2.0

Scenario 8

	d_1^B	d_2^B	d_3^B
d_1^A	3.2	2.1	1.5
d_2^A	2.4	8.4	**7.1**
d_3^A	**1.3**	4.6	5.3

Scenario 9

	d_1^B	d_2^B	d_3^B
d_1^A	5.0	5.9	**1.9**
d_2^A	**4.9**	8.9	3.3
d_3^A	0.9	2.3	1.8

Scenario 10

	d_1^B	d_2^B	d_3^B
d_1^A	7.7	**6.9**	1.1
d_2^A	**5.6**	7.0	1.8
d_3^A	0.7	1.6	0.6

Scenario 11

	d_1^B	d_2^B	d_3^B
d_1^A	4.5	5.5	3.9
d_2^A	**5.4**	8.2	3.6
d_3^A	1.1	1.6	1.5

Scenario 12

	d_1^B	d_2^B	d_3^B
d_1^A	8.9	**7.3**	1.1
d_2^A	3.9	5.0	1.5
d_3^A	0.4	0.6	0.3

Scenario 13

	d_1^B	d_2^B	d_3^B
d_1^A	**8.9**	3.9	0.4
d_2^A	3.5	3.8	0.8
d_3^A	0.3	0.6	0.3

Scenario 14

	d_1^B	d_2^B	d_3^B
d_1^A	6.4	2.6	0.2
d_2^A	2.3	2.0	0.3
d_3^A	0.2	0.2	0.1

Scenario 15

	d_1^B	d_2^B	d_3^B
d_1^A	3.0	1.5	1.0
d_2^A	1.6	1.3	1.8
d_3^A	1.0	1.8	22.8

Table 12: BLRM: Mean number of patients assigned to each dose combination. True MTCs are in bold and 'acceptable' combinations are underlined.

Scenario 1

	d_1^B	d_2^B	d_3^B
d_1^A	0.0	0.1	1.4
d_2^A	1.3	1.8	<u>6.2</u>
d_3^A	5.4	<u>8.2</u>	<u>**74.7**</u>

Scenario 2

	d_1^B	d_2^B	d_3^B
d_1^A	0.0	0.5	6.4
d_2^A	3.5	<u>5.1</u>	<u>**18.0**</u>
d_3^A	<u>11.2</u>	<u>**14.9**</u>	39.5

Scenario 3

	d_1^B	d_2^B	d_3^B
d_1^A	0.0	0.2	5.5
d_2^A	2.9	2.9	<u>16.4</u>
d_3^A	<u>7.6</u>	<u>**12.2**</u>	52.0

Scenario 4

	d_1^B	d_2^B	d_3^B
d_1^A	0.0	0.8	11.2
d_2^A	7.6	<u>11.6</u>	<u>**22.1**</u>
d_3^A	<u>13.8</u>	17.9	14.2

Scenario 5

	d_1^B	d_2^B	d_3^B
d_1^A	0.6	12.4	22.1
d_2^A	<u>15.8</u>	<u>**17.8**</u>	14.7
d_3^A	8.1	5.9	2.4

Scenario 6

	d_1^B	d_2^B	d_3^B
d_1^A	0.4	5.5	<u>**16.0**</u>
d_2^A	14.8	<u>**19.9**</u>	14.9
d_3^A	<u>**13.2**</u>	8.8	3.6

Scenario 7

	d_1^B	d_2^B	d_3^B
d_1^A	0.8	<u>6.8</u>	18.0
d_2^A	16.0	<u>**25.2**</u>	13.9
d_3^A	<u>**10.6**</u>	3.7	1.6

Scenario 8

	d_1^B	d_2^B	d_3^B
d_1^A	0.0	1.7	<u>15.5</u>
d_2^A	8.0	<u>11.9</u>	<u>**21.3**</u>
d_3^A	<u>**14.4**</u>	12.2	14.5

Scenario 9

	d_1^B	d_2^B	d_3^B
d_1^A	3.8	19.7	<u>**18.8**</u>
d_2^A	<u>**17.9**</u>	17.5	9.0
d_3^A	6.6	2.4	1.0

Scenario 10

	d_1^B	d_2^B	d_3^B
d_1^A	15.4	<u>**21.6**</u>	16.6
d_2^A	<u>**18.8**</u>	12.6	4.5
d_3^A	2.6	0.7	0.0

Scenario 11

	d_1^B	d_2^B	d_3^B
d_1^A	2.4	22.6	24.2
d_2^A	<u>**16.4**</u>	18.6	9.4
d_3^A	4.3	1.8	0.1

Scenario 12

	d_1^B	d_2^B	d_3^B
d_1^A	<u>38.1</u>	<u>**18.1**</u>	10.5
d_2^A	12.8	4.4	1.6
d_3^A	0.8	0.0	0.0

Scenario 13

	d_1^B	d_2^B	d_3^B
d_1^A	<u>**36.5**</u>	10.2	5.1
d_2^A	5.4	1.5	0.5
d_3^A	0.1	0.0	0.0

Scenario 14

	d_1^B	d_2^B	d_3^B
d_1^A	11.5	0.9	0.8
d_2^A	0.5	0.2	0.0
d_3^A	0.0	0.0	0.0

Scenario 15

	d_1^B	d_2^B	d_3^B
d_1^A	0.0	0.0	0.3
d_2^A	0.4	1.0	1.9
d_3^A	1.8	2.1	89.4

Table 13: POCRM: Percentage of simulations selecting each dose combination True MTCs are in bold and 'acceptable' combinations are underlined.

Scenario 1

	d_1^B	d_2^B	d_3^B
d_1^A	3.6	3.3	3.7
d_2^A	3.4	3.4	3.8
d_3^A	3.3	3.2	**8.1**

Scenario 2

	d_1^B	d_2^B	d_3^B
d_1^A	3.6	3.4	4.4
d_2^A	3.8	3.8	**4.7**
d_3^A	3.3	**3.3**	5.4

Scenario 3

	d_1^B	d_2^B	d_3^B
d_1^A	3.3	3.3	4.2
d_2^A	3.5	3.5	4.3
d_3^A	3.3	**3.2**	7.4

Scenario 4

	d_1^B	d_2^B	d_3^B
d_1^A	3.6	3.5	4.9
d_2^A	4.0	4.4	**5.4**
d_3^A	3.5	3.0	3.4

Scenario 5

	d_1^B	d_2^B	d_3^B
d_1^A	4.1	5.8	7.4
d_2^A	4.9	**5.7**	3.8
d_3^A	2.0	1.3	0.9

Scenario 6

	d_1^B	d_2^B	d_3^B
d_1^A	4.5	4.6	**6.2**
d_2^A	5.0	**5.3**	4.2
d_3^A	**2.6**	1.7	0.9

Scenario 7

	d_1^B	d_2^B	d_3^B
d_1^A	4.7	4.9	6.5
d_2^A	5.4	**5.6**	4.0
d_3^A	**2.2**	1.1	0.5

Scenario 8

	d_1^B	d_2^B	d_3^B
d_1^A	3.7	3.8	5.9
d_2^A	4.3	4.7	**5.1**
d_3^A	**3.1**	2.5	2.8

Scenario 9

	d_1^B	d_2^B	d_3^B
d_1^A	6.6	7.7	**6.5**
d_2^A	**5.0**	4.6	2.4
d_3^A	1.2	0.7	0.3

Scenario 10

	d_1^B	d_2^B	d_3^B
d_1^A	9.0	**8.2**	6.2
d_2^A	**5.2**	3.1	1.3
d_3^A	0.5	0.2	0.0

Scenario 11

	d_1^B	d_2^B	d_3^B
d_1^A	5.0	8.2	7.4
d_2^A	**5.2**	5.4	2.8
d_3^A	1.2	0.6	0.2

Scenario 12

	d_1^B	d_2^B	d_3^B
d_1^A	15.9	**7.9**	3.8
d_2^A	2.9	1.2	0.3
d_3^A	0.1	0.0	0.0

Scenario 13

	d_1^B	d_2^B	d_3^B
d_1^A	**15.6**	4.7	2.4
d_2^A	1.9	0.7	0.2
d_3^A	0.1	0.0	0.0

Scenario 14

	d_1^B	d_2^B	d_3^B
d_1^A	10.9	1.5	0.7
d_2^A	0.6	0.2	0.1
d_3^A	0.0	0.0	0.0

Scenario 15

	d_1^B	d_2^B	d_3^B
d_1^A	4.1	3.3	3.3
d_2^A	3.2	3.1	3.1
d_3^A	2.9	2.8	9.3

Table 14: POCRM: Mean number of patients assigned to each dose combination. True MTCs are in bold and ‘acceptable’ combinations are underlined.

Scenario 1

	d_1^B	d_2^B	d_3^B
d_1^A	0.0	0.2	0.7
d_2^A	0.0	3.8	11.4
d_3^A	0.4	14.6	**68.0**

Scenario 2

	d_1^B	d_2^B	d_3^B
d_1^A	0.0	0.9	2.6
d_2^A	0.9	19.4	**29.6**
d_3^A	0.8	**25.4**	18.9

Scenario 3

	d_1^B	d_2^B	d_3^B
d_1^A	0.0	0.4	5.7
d_2^A	0.0	8.1	35.9
d_3^A	0.2	**22.5**	27.3

Scenario 4

	d_1^B	d_2^B	d_3^B
d_1^A	0.0	1.0	14.0
d_2^A	0.4	32.2	**21.5**
d_3^A	0.8	26.6	2.6

Scenario 5

	d_1^B	d_2^B	d_3^B
d_1^A	0.1	17.0	5.1
d_2^A	0.7	**59.5**	2.8
d_3^A	1.6	13.2	0.1

Scenario 6

	d_1^B	d_2^B	d_3^B
d_1^A	0.3	9.4	**7.8**
d_2^A	5.8	**51.4**	9.3
d_3^A	**2.8**	9.1	0.8

Scenario 7

	d_1^B	d_2^B	d_3^B
d_1^A	0.2	9.6	8.5
d_2^A	15.2	**50.6**	5.6
d_3^A	**1.8**	5.6	0.4

Scenario 8

	d_1^B	d_2^B	d_3^B
d_1^A	0.0	2.0	12.8
d_2^A	0.8	32.5	**17.7**
d_3^A	**0.6**	28.2	4.4

Scenario 9

	d_1^B	d_2^B	d_3^B
d_1^A	1.8	31.9	**5.5**
d_2^A	**12.4**	30.2	2.8
d_3^A	9.7	2.6	0.2

Scenario 10

	d_1^B	d_2^B	d_3^B
d_1^A	9.1	**35.0**	7.2
d_2^A	**22.1**	14.3	0.7
d_3^A	3.8	0.4	0.0

Scenario 11

	d_1^B	d_2^B	d_3^B
d_1^A	0.4	56.8	3.7
d_2^A	**0.4**	33.8	0.7
d_3^A	2.8	1.3	0.0

Scenario 12

	d_1^B	d_2^B	d_3^B
d_1^A	25.0	**21.3**	0.6
d_2^A	29.8	3.1	0.0
d_3^A	6.3	0.4	0.0

Scenario 13

	d_1^B	d_2^B	d_3^B
d_1^A	**35.0**	12.8	1.2
d_2^A	7.2	2.0	0.0
d_3^A	0.8	0.1	0.1

Scenario 14

	d_1^B	d_2^B	d_3^B
d_1^A	10.2	2.4	0.4
d_2^A	1.8	0.2	0.0
d_3^A	0.0	0.0	0.0

Scenario 15

	d_1^B	d_2^B	d_3^B
d_1^A	2.0	0.0	0.0
d_2^A	0.0	0.4	0.6
d_3^A	0.2	1.6	92.0

Table 15: Riviere: Percentage of simulations selecting each dose combination True MTCs are in bold and 'acceptable' combinations are underlined.

Scenario 1

	d_1^B	d_2^B	d_3^B
d_1^A	3.5	2.5	1.8
d_2^A	1.0	2.7	5.1
d_3^A	0.6	4.3	**14.2**

Scenario 2

	d_1^B	d_2^B	d_3^B
d_1^A	3.5	2.8	2.4
d_2^A	1.2	4.7	**7.0**
d_3^A	1.1	**6.1**	6.7

Scenario 3

	d_1^B	d_2^B	d_3^B
d_1^A	3.2	2.8	2.6
d_2^A	0.7	3.7	8.4
d_3^A	0.7	**5.7**	8.1

Scenario 4

	d_1^B	d_2^B	d_3^B
d_1^A	3.5	2.8	3.5
d_2^A	1.2	6.0	**7.7**
d_3^A	1.5	6.3	3.2

Scenario 5

	d_1^B	d_2^B	d_3^B
d_1^A	3.2	5.2	3.6
d_2^A	1.8	**11.0**	3.5
d_3^A	2.2	4.7	0.8

Scenario 6

	d_1^B	d_2^B	d_3^B
d_1^A	4.1	3.8	**3.5**
d_2^A	2.6	**8.8**	4.5
d_3^A	**2.3**	4.0	1.5

Scenario 7

	d_1^B	d_2^B	d_3^B
d_1^A	4.2	3.8	3.9
d_2^A	3.8	**9.0**	4.0
d_3^A	**2.3**	3.2	1.1

Scenario 8

	d_1^B	d_2^B	d_3^B
d_1^A	3.5	3.0	3.7
d_2^A	1.3	6.4	**7.0**
d_3^A	**1.5**	6.3	3.1

Scenario 9

	d_1^B	d_2^B	d_3^B
d_1^A	4.5	6.3	**3.2**
d_2^A	**4.4**	8.4	2.0
d_3^A	3.6	2.0	0.6

Scenario 10

	d_1^B	d_2^B	d_3^B
d_1^A	6.3	**7.2**	3.7
d_2^A	**6.1**	5.8	1.2
d_3^A	2.2	0.9	0.3

Scenario 11

	d_1^B	d_2^B	d_3^B
d_1^A	3.3	9.2	2.9
d_2^A	**2.8**	11.1	1.5
d_3^A	3.1	1.8	0.3

Scenario 12

	d_1^B	d_2^B	d_3^B
d_1^A	9.0	**6.9**	1.6
d_2^A	7.9	3.1	0.3
d_3^A	2.8	0.4	0.1

Scenario 13

	d_1^B	d_2^B	d_3^B
d_1^A	**11.5**	5.3	1.4
d_2^A	4.1	1.6	0.3
d_3^A	0.8	0.2	0.1

Scenario 14

	d_1^B	d_2^B	d_3^B
d_1^A	8.8	1.8	0.4
d_2^A	1.5	0.5	0.1
d_3^A	0.3	0.0	0.0

Scenario 15

	d_1^B	d_2^B	d_3^B
d_1^A	4.0	2.3	1.4
d_2^A	1.1	2.0	2.4
d_3^A	0.5	1.5	19.8

Table 16: Riviere: Mean number of patients assigned to each dose combination. True MTCs are in bold and 'acceptable' combinations are underlined.

Scenario 16

	d_1^B	d_2^B	d_3^B
d_1^A	18.6	**29.2**	5.9
d_2^A	**33.2**	9.3	0.2

Scenario 17

	d_1^B	d_2^B	d_3^B
d_1^A	4.1	25.4	**31.0**
d_2^A	**25.9**	9.6	0.4

Scenario 18

	d_1^B	d_2^B	d_3^B
d_1^A	1.6	9.6	**16.2**
d_2^A	27.2	**37.9**	4.8

Scenario 19

	d_1^B	d_2^B	d_3^B	d_4^B
d_1^A	4.6	9.6	16.9	**23.9**
d_2^A	**23.9**	13.6	3.8	0.2

Scenario 20

	d_1^B	d_2^B	d_3^B	d_4^B
d_1^A	3.4	9.8	**11.5**	5.7
d_2^A	24.6	**28.5**	11.7	2.4

Scenario 21

	d_1^B	d_2^B	d_3^B	d_4^B
d_1^A	13.1	17.2	17.9	**20.2**
d_2^A	14.4	5.8	2.2	0.4

Table 17: Alternative dosing grid: BOIN: Percentage of simulations selecting each dose combination. True MTCs are in bold and ‘acceptable’ combinations are underlined.

Scenario 16

	d_1^B	d_2^B	d_3^B
d_1^A	8.6	<u>**8.1**</u>	2.8
d_2^A	<u>**10.0**</u>	4.5	0.8

Scenario 17

	d_1^B	d_2^B	d_3^B
d_1^A	5.7	7.2	<u>**7.7**</u>
d_2^A	7.9	<u>**5.1**</u>	1.3

Scenario 18

	d_1^B	d_2^B	d_3^B
d_1^A	4.7	<u>4.4</u>	<u>**4.0**</u>
d_2^A	8.2	<u>**10.1**</u>	3.6

Scenario 19

	d_1^B	d_2^B	d_3^B	d_4^B
d_1^A	5.7	4.8	<u>4.4</u>	<u>**4.4**</u>
d_2^A	<u>**7.5**</u>	5.4	2.0	0.5

Scenario 20

	d_1^B	d_2^B	d_3^B	d_4^B
d_1^A	5.3	<u>4.7</u>	<u>**3.0**</u>	1.4
d_2^A	<u>7.9</u>	<u>**8.0**</u>	3.8	1.1

Scenario 21

	d_1^B	d_2^B	d_3^B	d_4^B
d_1^A	8.2	<u>6.2</u>	<u>4.4</u>	<u>**3.6**</u>
d_2^A	6.0	3.3	1.1	0.4

Table 18: Alternative dosing grid: BOIN: Mean number of patients assigned to each dose combination. True MTCs are in bold and 'acceptable' combinations are underlined.

Scenario 16

	d_1^B	d_2^B	d_3^B
d_1^A	16.4	<u>**30.2**</u>	6.9
d_2^A	<u>**35.9**</u>	8.5	0.2

Scenario 17

	d_1^B	d_2^B	d_3^B
d_1^A	4.0	<u>**22.9**</u>	31.9
d_2^A	<u>**28.6**</u>	10.8	0.5

Scenario 18

	d_1^B	d_2^B	d_3^B
d_1^A	1.6	<u>11.1</u>	<u>**18.0**</u>
d_2^A	23.5	<u>**39.0**</u>	5.3

Scenario 19

	d_1^B	d_2^B	d_3^B	d_4^B
d_1^A	4.0	11.5	<u>19.9</u>	<u>**20.4**</u>
d_2^A	<u>**25.8**</u>	13.8	3.2	0.2

Scenario 20

	d_1^B	d_2^B	d_3^B	d_4^B
d_1^A	2.5	<u>12.0</u>	<u>**12.0**</u>	5.8
d_2^A	<u>23.6</u>	<u>**29.5**</u>	11.0	1.6

Scenario 21

	d_1^B	d_2^B	d_3^B	d_4^B
d_1^A	14.8	<u>20.4</u>	<u>18.1</u>	<u>**16.2**</u>
d_2^A	14.8	6.5	3.0	0.6

Table 19: Alternative dosing grid: Keyboard: Percentage of simulations selecting each dose combination. True MTCs are in bold and 'acceptable' combinations are underlined.

Scenario 16

	d_1^B	d_2^B	d_3^B
d_1^A	8.7	<u>**8.9**</u>	2.5
d_2^A	<u>**10.5**</u>	4.2	0.7

Scenario 17

	d_1^B	d_2^B	d_3^B
d_1^A	6.3	6.9	<u>**6.9**</u>
d_2^A	<u>**8.7**</u>	5.5	1.4

Scenario 18

	d_1^B	d_2^B	d_3^B
d_1^A	5.3	<u>5.4</u>	<u>**4.1**</u>
d_2^A	7.1	<u>**10.0**</u>	3.6

Scenario 19

	d_1^B	d_2^B	d_3^B	d_4^B
d_1^A	6.4	5.5	<u>4.4</u>	<u>**3.3**</u>
d_2^A	<u>**8.2**</u>	5.5	1.9	0.4

Scenario 20

	d_1^B	d_2^B	d_3^B	d_4^B
d_1^A	5.7	<u>5.5</u>	<u>**3.0**</u>	1.1
d_2^A	<u>7.5</u>	<u>**8.0**</u>	3.7	1.0

Scenario 21

	d_1^B	d_2^B	d_3^B	d_4^B
d_1^A	9.9	<u>6.8</u>	<u>4.0</u>	<u>**2.6**</u>
d_2^A	6.2	3.3	1.2	0.4

Table 20: Alternative dosing grid: Keyboard: Mean number of patients assigned to each dose combination. True MTCs are in bold and 'acceptable' combinations are underlined.

Scenario 16

	d_1^B	d_2^B	d_3^B
d_1^A	2.5	**<u>37.3</u>**	7.5
d_2^A	**<u>37.4</u>**	11.1	0.3

Scenario 17

	d_1^B	d_2^B	d_3^B
d_1^A	0.8	**<u>16.0</u>**	30.2
d_2^A	**<u>29.6</u>**	18.0	1.6

Scenario 18

	d_1^B	d_2^B	d_3^B
d_1^A	0.3	<u>18.2</u>	**<u>21.4</u>**
d_2^A	11.6	**<u>35.5</u>**	9.2

Scenario 19

	d_1^B	d_2^B	d_3^B	d_4^B
d_1^A	0.7	10.8	<u>22.9</u>	**<u>12.2</u>**
d_2^A	**<u>24.9</u>**	19.2	5.5	0.4

Scenario 20

	d_1^B	d_2^B	d_3^B	d_4^B
d_1^A	0.5	<u>15.4</u>	**<u>19.1</u>**	3.1
d_2^A	<u>15.2</u>	**<u>31.0</u>**	11.4	0.9

Scenario 21

	d_1^B	d_2^B	d_3^B	d_4^B
d_1^A	4.5	<u>20.6</u>	<u>22.7</u>	**<u>12.4</u>**
d_2^A	21.1	7.4	2.0	0.1

Table 21: Alternative dosing grid: SFD: Percentage of simulations selecting each dose combination. True MTCs are in bold and ‘acceptable’ combinations are underlined.

Scenario 16

	d_1^B	d_2^B	d_3^B
d_1^A	7.7	$\underline{\mathbf{9.7}}$	2.5
d_2^A	$\underline{\mathbf{10.2}}$	4.4	0.2

Scenario 17

	d_1^B	d_2^B	d_3^B
d_1^A	5.0	$\underline{\mathbf{8.4}}$	6.8
d_2^A	$\underline{\mathbf{7.9}}$	6.0	0.7

Scenario 18

	d_1^B	d_2^B	d_3^B
d_1^A	4.3	$\underline{6.2}$	$\underline{\mathbf{5.4}}$
d_2^A	6.9	$\underline{\mathbf{9.6}}$	2.4

Scenario 19

	d_1^B	d_2^B	d_3^B	d_4^B
d_1^A	5.0	7.1	$\underline{5.8}$	$\underline{\mathbf{2.0}}$
d_2^A	$\underline{\mathbf{7.3}}$	6.2	1.5	0.1

Scenario 20

	d_1^B	d_2^B	d_3^B	d_4^B
d_1^A	4.6	$\underline{6.4}$	$\underline{\mathbf{4.9}}$	0.8
d_2^A	$\underline{7.2}$	$\underline{\mathbf{8.6}}$	2.3	0.2

Scenario 21

	d_1^B	d_2^B	d_3^B	d_4^B
d_1^A	7.1	$\underline{8.7}$	$\underline{5.2}$	$\underline{\mathbf{1.9}}$
d_2^A	6.4	3.3	0.7	0.1

Table 22: Alternative dosing grid: SFD: Mean number of patients assigned to each dose combination. True MTCs are in bold and 'acceptable' combinations are underlined.

Scenario 16

	d_1^B	d_2^B	d_3^B
d_1^A	46.5	**28.8**	0.8
d_2^A	**20.4**	0.3	0.0

Scenario 17

	d_1^B	d_2^B	d_3^B
d_1^A	9.0	53.8	**5.4**
d_2^A	**26.4**	1.7	0.0

Scenario 18

	d_1^B	d_2^B	d_3^B
d_1^A	10.8	30.4	**10.9**
d_2^A	31.9	**12.2**	0.0

Scenario 19

	d_1^B	d_2^B	d_3^B	d_4^B
d_1^A	7.3	15.7	41.0	**0.8**
d_2^A	**30.5**	3.1	0.1	0.0

Scenario 20

	d_1^B	d_2^B	d_3^B	d_4^B
d_1^A	9.4	25.9	**20.9**	0.4
d_2^A	31.4	**8.6**	0.4	0.0

Scenario 21

	d_1^B	d_2^B	d_3^B	d_4^B
d_1^A	16.8	27.9	30.7	**0.1**
d_2^A	18.1	1.0	0.0	0.0

Table 23: Alternative dosing grid: PIPE: Percentage of simulations selecting each dose combination. True MTCs are in bold and 'acceptable' combinations are underlined.

Scenario 16

	d_1^B	d_2^B	d_3^B
d_1^A	12.9	<u>**12.3**</u>	0.7
d_2^A	<u>**8.7**</u>	0.7	0.0

Scenario 17

	d_1^B	d_2^B	d_3^B
d_1^A	5.4	16.9	<u>**2.0**</u>
d_2^A	<u>**8.9**</u>	1.6	0.0

Scenario 18

	d_1^B	d_2^B	d_3^B
d_1^A	5.8	<u>11.1</u>	<u>**3.6**</u>
d_2^A	10.7	<u>**3.7**</u>	0.0

Scenario 19

	d_1^B	d_2^B	d_3^B	d_4^B
d_1^A	4.6	6.8	<u>17.0</u>	<u>**0.2**</u>
d_2^A	<u>**5.1**</u>	1.9	0.1	0.0

Scenario 20

	d_1^B	d_2^B	d_3^B	d_4^B
d_1^A	4.9	<u>7.9</u>	<u>**11.0**</u>	0.3
d_2^A	<u>7.5</u>	<u>**3.6**</u>	0.2	0.0

Scenario 21

	d_1^B	d_2^B	d_3^B	d_4^B
d_1^A	6.4	<u>8.8</u>	<u>13.7</u>	<u>**0.0**</u>
d_2^A	4.9	1.0	0.0	0.0

Table 24: Alternative dosing grid: PIPE: Mean number of patients assigned to each dose combination. True MTCs are in bold and 'acceptable' combinations are underlined.

Scenario 16

	d_1^B	d_2^B	d_3^B
d_1^A	21.5	**26.6**	11.2
d_2^A	**27.8**	8.4	0.4

Scenario 17

	d_1^B	d_2^B	d_3^B
d_1^A	8.8	18.8	**33.4**
d_2^A	**27.2**	7.2	0.4

Scenario 18

	d_1^B	d_2^B	d_3^B
d_1^A	6.8	17.0	**30.**
d_2^A	12.4	**24.6**	4.6

Scenario 19

	d_1^B	d_2^B	d_3^B	d_4^B
d_1^A	8.6	8.8	20.1	**21.9**
d_2^A	**24.9**	8.8	2.7	0.1

Scenario 20

	d_1^B	d_2^B	d_3^B	d_4^B
d_1^A	7.4	15.4	**19.1**	11.2
d_2^A	15.8	**18.1**	7.5	1.2

Scenario 21

	d_1^B	d_2^B	d_3^B	d_4^B
d_1^A	21.6	14.5	18.6	**15.4**
d_2^A	15.3	3.3	1.0	0.0

Table 25: Alternative dosing grid: Waterfall: Percentage of simulations selecting each dose combination. True MTCs are in bold and 'acceptable' combinations are underlined.

Scenario 16

	d_1^B	d_2^B	d_3^B
d_1^A	6.8	**4.7**	3.0
d_2^A	**7.5**	3.3	0.6

Scenario 17

	d_1^B	d_2^B	d_3^B
d_1^A	6.8	4.5	**5.7**
d_2^A	**7.5**	3.3	0.6

Scenario 18

	d_1^B	d_2^B	d_3^B
d_1^A	4.6	3.4	**6.7**
d_2^A	6.5	**6.5**	2.7

Scenario 19

	d_1^B	d_2^B	d_3^B	d_4^B
d_1^A	6.8	3.5	3.8	**2.9**
d_2^A	**7.1**	3.4	1.0	0.1

Scenario 20

	d_1^B	d_2^B	d_3^B	d_4^B
d_1^A	5.1	3.2	**3.9**	2.7
d_2^A	6.5	**5.1**	2.4	0.7

Scenario 21

	d_1^B	d_2^B	d_3^B	d_4^B
d_1^A	8.2	3.7	2.9	**1.6**
d_2^A	5.8	1.8	0.4	0.1

Table 26: Alternative dosing grid: Waterfall: Mean number of patients assigned to each dose combination. True MTCs are in bold and 'acceptable' combinations are underlined.

Scenario 16

	d_1^B	d_2^B	d_3^B
d_1^A	8.0	**32.5**	5.3
d_2^A	**17.9**	13.4	0.6

Scenario 17

	d_1^B	d_2^B	d_3^B
d_1^A	1.6	19.4	**20.9**
d_2^A	**19.9**	17.4	1.3

Scenario 18

	d_1^B	d_2^B	d_3^B
d_1^A	0.3	9.4	**6.3**
d_2^A	6.3	**53.7**	11.2

Scenario 19

	d_1^B	d_2^B	d_3^B	d_4^B
d_1^A	1.7	5.6	21.6	**13.2**
d_2^A	**12.7**	26.4	6.5	0.8

Scenario 20

	d_1^B	d_2^B	d_3^B	d_4^B
d_1^A	1.3	6.3	**11.1**	2.6
d_2^A	5.1	**36.6**	25.2	5.2

Scenario 21

	d_1^B	d_2^B	d_3^B	d_4^B
d_1^A	3.8	12.1	21.4	**14.6**
d_2^A	10.3	17.5	5.0	0.7

Table 27: Alternative dosing grid: BLRM: Percentage of simulations selecting each dose combination. True MTCs are in bold and 'acceptable' combinations are underlined.

Scenario 16

	d_1^B	d_2^B	d_3^B
d_1^A	5.4	**5.4**	2.6
d_2^A	**6.0**	8.0	4.9

Scenario 17

	d_1^B	d_2^B	d_3^B
d_1^A	4.5	4.1	**5.3**
d_2^A	**5.8**	8.1	5.1

Scenario 18

	d_1^B	d_2^B	d_3^B
d_1^A	3.7	1.7	**1.7**
d_2^A	4.2	**12.0**	10.9

Scenario 19

	d_1^B	d_2^B	d_3^B	d_4^B
d_1^A	4.4	1.2	3.9	**3.3**
d_2^A	**4.3**	7.4	6.5	3.4

Scenario 20

	d_1^B	d_2^B	d_3^B	d_4^B
d_1^A	3.6	0.9	**1.9**	0.9
d_2^A	3.7	**7.8**	9.4	6.9

Scenario 21

	d_1^B	d_2^B	d_3^B	d_4^B
d_1^A	5.1	2.3	4.3	**3.7**
d_2^A	4.1	6.5	5.2	2.7

Table 28: Alternative dosing grid: BLRM: Mean number of patients assigned to each dose combination. True MTCs are in bold and 'acceptable' combinations are underlined.

Scenario 16

	d_1^B	d_2^B	d_3^B
d_1^A	15.0	<u>**28.8**</u>	24.4
d_2^A	<u>**17.9**</u>	8.2	2.2

Scenario 17

	d_1^B	d_2^B	d_3^B
d_1^A	3.4	14.8	<u>**26.7**</u>
d_2^A	<u>**28.0**</u>	18.1	5.7

Scenario 18

	d_1^B	d_2^B	d_3^B
d_1^A	1.3	<u>9.8</u>	<u>**24.2**</u>
d_2^A	18.0	<u>**20.9**</u>	23.0

Scenario 19

	d_1^B	d_2^B	d_3^B	d_4^B
d_1^A	0.7	4.1	<u>13.4</u>	<u>**24.8**</u>
d_2^A	<u>**23.8**</u>	18.6	8.7	3.1

Scenario 20

	d_1^B	d_2^B	d_3^B	d_4^B
d_1^A	2.3	<u>8.9</u>	<u>**16.9**</u>	22.4
d_2^A	<u>16.3</u>	<u>**12.1**</u>	10.2	8.3

Scenario 21

	d_1^B	d_2^B	d_3^B	d_4^B
d_1^A	4.3	<u>9.6</u>	<u>19.4</u>	<u>**24.3**</u>
d_2^A	19.1	9.1	4.6	2.4

Table 29: Alternative dosing grid: POCRM: Percentage of simulations selecting each dose combination. True MTCs are in bold and 'acceptable' combinations are underlined.

Scenario 16

	d_1^B	d_2^B	d_3^B
d_1^A	8.8	**<u>9.1</u>**	7.7
d_2^A	**<u>5.0</u>**	2.8	1.6

Scenario 17

	d_1^B	d_2^B	d_3^B
d_1^A	5.6	5.9	**<u>8.4</u>**
d_2^A	**<u>6.8</u>**	4.8	3.5

Scenario 18

	d_1^B	d_2^B	d_3^B
d_1^A	5.4	<u>5.3</u>	**<u>7.3</u>**
d_2^A	5.1	**<u>4.9</u>**	7.1

Scenario 19

	d_1^B	d_2^B	d_3^B	d_4^B
d_1^A	4.7	4.3	<u>5.8</u>	**<u>6.7</u>**
d_2^A	**<u>5.4</u>**	4.1	2.4	1.6

Scenario 20

	d_1^B	d_2^B	d_3^B	d_4^B
d_1^A	5.6	<u>5.5</u>	**<u>7.1</u>**	6.1
d_2^A	<u>3.8</u>	**<u>3.0</u>**	1.9	2.1

Scenario 21

	d_1^B	d_2^B	d_3^B	d_4^B
d_1^A	6.5	<u>5.1</u>	<u>6.4</u>	**<u>6.2</u>**
d_2^A	4.7	2.8	1.2	0.8

Table 30: Alternative dosing grid: POCRM: Mean number of patients assigned to each dose combination. True MTCs are in bold and 'acceptable' combinations are underlined.

Scenario 16

	d_1^B	d_2^B	d_3^B
d_1^A	5.5	**22.4**	49.5
d_2^A	**13.4**	6.3	0.2

Scenario 17

	d_1^B	d_2^B	d_3^B
d_1^A	1.3	23.3	**24.3**
d_2^A	**25.9**	20.8	1.8

Scenario 18

	d_1^B	d_2^B	d_3^B
d_1^A	0.4	5.0	**17.5**
d_2^A	45.7	**15.0**	13.1

Scenario 19

	d_1^B	d_2^B	d_3^B	d_4^B
d_1^A	0.8	12.6	10.2	**26.2**
d_2^A	**21.6**	9.4	15.6	0.8

Scenario 20

	d_1^B	d_2^B	d_3^B	d_4^B
d_1^A	0.6	5.0	**16.4**	26.1
d_2^A	23.0	**19.1**	4.2	3.3

Scenario 21

	d_1^B	d_2^B	d_3^B	d_4^B
d_1^A	5.1	12.8	20.4	**12.7**
d_2^A	19.7	5.0	16.1	0.6

Table 31: Alternative dosing grid: Riviere: Percentage of simulations selecting each dose combination. True MTCs are in bold and 'acceptable' combinations are underlined.

Scenario 16

	d_1^B	d_2^B	d_3^B
d_1^A	5.7	**6.4**	10.5
d_2^A	**6.4**	4.6	1.5

Scenario 17

	d_1^B	d_2^B	d_3^B
d_1^A	4.5	5.6	**6.6**
d_2^A	**9.4**	6.7	2.4

Scenario 18

	d_1^B	d_2^B	d_3^B
d_1^A	4.3	3.5	**4.8**
d_2^A	9.9	**6.2**	6.3

Scenario 19

	d_1^B	d_2^B	d_3^B	d_4^B
d_1^A	4.5	4.1	4.3	**5.9**
d_2^A	**5.6**	5.1	4.6	1.0

Scenario 20

	d_1^B	d_2^B	d_3^B	d_4^B
d_1^A	4.4	3.6	**4.7**	6.1
d_2^A	5.6	**5.2**	3.2	2.4

Scenario 21

	d_1^B	d_2^B	d_3^B	d_4^B
d_1^A	6.0	4.6	5.8	**4.0**
d_2^A	5.4	3.3	3.8	0.7

Table 32: Alternative dosing grid: Riviere: Mean number of patients assigned to each dose combination. True MTCs are in bold and 'acceptable' combinations are underlined.

5 Summary of Hyper-parameters

	Calibrated	Original Recommendation
**BOIN**	$a_1 = 0.65$<br>$a_2 = 1.4$	$a_1 = 0.6$<br>$a_2 = 1.4$
**Keyboard**	$b_1 = 0.21$<br>$b_2 = 0.39$	$b_1 = 0.25$<br>$b_2 = 0.35$
**Surface-Free**	$m = 0.875$<br>$s_{\text{SFD}} = 4$	$m = 0.9525$<br>$s_{\text{SFD}} = 4$
**PIPE**	$s_{\text{PIPE}} = 1/18$<br>$\rho = 0.05$<br>$\delta = 0.025$	$s_{\text{PIPE}} = 1/9$<br>$\rho = 0.02$<br>$\delta \in [0.01, 0.07]$
**Waterfall**	$a_1 = 0.65$<br>$a_2 = 1.4$	$a_1 = 0.6$<br>$a_2 = 1.4$
**BLRM**	$c_1 = -2.5$<br>$c_2 = 0$<br>$v_1 = 1$<br>$v_2 = 0.5$<br>$v_3 = 0.03$	$c_1 = log(0.25)$<br>$c_2 = 0$<br>$v_1 = 4$<br>$v_2 = 1$<br>$v_3 = 0.03$
**POCRM**	$\delta = 0.015$<br>$\sigma^2 = 2.84$	$\tilde{q} = (0.04, 0.06, 0.10, 0.14, 0.19, 0.24, 0.30, 0.36, 0.42)$
**Riviere**	$a = 4800$<br>$b = 0.35$<br>$c = 2.5$	$a = 400$<br>$b = 1$<br>$c = 10$

Table 33: The values of hyper-parameters resulting from the prior calibration procedure and from original recommendations.

References

[1] Shing M Lee and Ying Kuen Cheung. Model calibration in the continual reassessment method. *Clinical Trials*, 6(3):227–238, 2009. 3

[2] Shing M Lee and Ying Kuen Cheung. Calibration of prior variance in the bayesian continual reassessment method. *Statistics in medicine*, 30(17):2081–2089, 2011. 4

[3] Pavel Mozgunov, Rochelle Knight, Helen Barnett, and Thomas Jaki. Using an interaction parameter in model-based phase i trials for combination treatments? a simulation study. *International journal of environmental research and public health*, 18(1):345, 2021. 4

[4] Beat Neuenschwander, Alessandro Matano, Zhongwen Tang, Satrajit Roychoudhury, Simon Wandel, and Stuart Bailey. A Bayesian industry approach to phase I combination trials in oncology. In *Statistical methods in drug combination studies*, pages 95–135. Chapman & Hall/CRC Press: Boca Raton, FL, 2015. 3
